# Elasticity standard values of the Achilles tendon assessed with acoustic radiation force impulse elastography on healthy volunteers: a cross section study

**DOI:** 10.1186/s12891-018-2056-0

**Published:** 2018-05-09

**Authors:** Jonas Wakker, Wolfgang Kratzer, Tilmann Graeter, Julian Schmidberger, PhD for the Elasto Study Group, Anne Heizelmann, Simon Boretzki, Sarah Uhlemann, Sabrina Galgenmüller, Sabina Stumpf, Judith Schulze, Christine Neuwirt, Beryl Emrich, Heike Jäger

**Affiliations:** 1 Department of General, Thoracic and Vascular Surgery, Triemli City Hospital, Birmensdorferstrasse 497, 8063 Zurich, Switzerland; 2 Department of Internal Medicine I, University Hospital Ulm, Albert-Einstein-Allee 23, 89081 Ulm, Germany; 3 Department of Interventional and Diagnostic Radiology, University Hospital Ulm, Albert-Einstein-Allee 23, 89081 Ulm, Germany

**Keywords:** ARFI, Elastography, Achilles tendon, Shear wave elastography, VTIQ

## Abstract

**Background:**

To determine normal values for acoustic radiation force impulse (ARFI) shear wave elastography of the Achilles tendon.

**Methods:**

Using the VTIQ mode with the Acuson S3000™ (Siemens Healthcare, Erlangen, Germany), we measured the shear wave velocity (SWV) in m/s and the diameter in mm of both Achilles tendons in 182 healthy volunteers. The tendon was displayed in a sagittal view with a relaxed tendon. The parameters were tested for correlations with the anthropometric data of the subjects, between the genders and different age groups, as well as information obtained from the history, such as smoking and sporting activities.

**Results:**

Using a sagittal acoustic window, we determined a mean SWV of 9.09 ± 0.71 m/s for the left Achilles tendon and 9.17 ± 0.61 m/s for the right. There was a significant difference between the results for the right and left side (*p* < 0.05). The diameter on the left was 4.7 ± 0.9 mm. On the right, it was 4.8 ± 0.9 mm. Likewise there was a significant difference between the results for the diameter of the left and right side (*p* < 0.05). Neither gender, body mass index (BMI) nor age had a significant effect on either of the measured parameters (*p* > 0.05). The same goes for the consumption of tobacco and alcohol (*p* > 0.05).

**Conclusions:**

Age, gender, BMI, smoking or the consumption of alcohol did not affect either the elasticity or the diameter of the Achilles tendon.

## Background

Although elastography was originally developed with the aim of improving the characterisation of tumors, this technique is being used increasingly in the diagnosis of musculoskeletal diseases. The Achilles tendon, thickest and strongest tendon in the human body, is the most frequently investigated tendon in ultrasound- studies. The Tendon can be affected by degenerative diseases like tendinopathies or rupture due to trauma.

Musculoskeletal diseases, especially the ones affecting the Achilles tendon, have a mayor financial impact. Tendinopathies, together with partial or complete tendon tears, are the reasons for almost one in ten visits to the doctor and are responsible for half of all the sports injuries diagnosed [1]. For a long time, conventional B-mode ultrasonography has been used to document changes in the tendons. Thickening, gaps and loss of echotexture in patients with tendinopathies can be recognised in direct comparison with healthy study populations [2].

In elastographic investigations, healthy tendons can be seen as firm structures [3]. The tendons in Patients with tendinopathy or with a rupture appeared considerably softer [4, 5].

Ooi et al. [6] summarised the above-mentioned studies and concluded that elastography can provide solid information on the healing process in tendons. Recommendations for returning to sporting activities after tendon injuries can also be based on the results.

Elastography should not, however, be considered a substitute for B-mode examinations of musculoskeletal structures. It rather could be included as additional imaging in the diagnostic algorithm and used, for example, to monitor post-traumatic healing processes or to allow the postoperative assessment of load-bearing capacity.

One mayor concern with the studies mentioned above is, that there is a copious variety of different ultrasound units being used to generate those elastographic measurements. These studies are very heterogeneous with respect to the technique used and the presentation of the elastographic properties.

Elasticity can be presented qualitatively in the form of coloured or black/grey/white elastograms or quantitatively in kilopascal (kPa) or metres per second (m/s). The elastographic measurements generated on different setups are therefore often not comparable [5].

There are some elastographic applications, such as real-time elastography or transient elastography, in which the examiner initiates the measurement by applying pressure with the transducer. One example where the transducer itself triggers the mechanical impulse is the acoustic radiation force impulse (ARFI) technique, employed in the Aixplorer® from Supersonic Imaging and the Acuson S3000™ from Siemens.

To the best of our knowledge, only one study on normal values for the Achilles tendon with the Acuson S3000 has so far been published. In the present paper, we would like to compare our results with this earlier study by Fu et al. (2016) and provide normal values to distinguish tendons with pathological results in routine clinical practice [7].

## Methods

### Study population

For this prospective study, we recruited subjects between November 2014 and April 2015. Previous medical conditions, surgery on the back or lower limbs, and pregnancy were exclusion criteria. Any pathological changes seen on B-mode scanning were also reasons for exclusion. Participants also had to be over the age of 18 years. The study was conducted in the agreement with the Declaration of Helsinki and with the approval of the Ethics Committee of the State Medical Chamber of Baden-Württemberg. Registration number: 07/15.

### Study protocol

Measurements on the Achilles tendon were made with the subjects lying prone, with the foot relaxed and hanging over the lower edge of the examination table. For the elastographic measurements, the patient was neither plantar flexed nor dorsiflexed at the ankle joint, so that the Achilles tendon was in a relaxed state. The tendon was preliminary examined with conventional B-Mode sonography. During this time, the subject was able to accustom to the examination and make sure that the lower extremity was completely relaxed. The examiner always informed the study participant about the correct positioning. The measurements were only accomplished when the examiner was able to depict the relaxed tendon according to the study protocol.

No manual pressure was to be applied to the transducer during the examination, allowing only its own weight to be used. Ultrasound gel was used to maintain good contact between skin and probe.

**Fig. 1:**
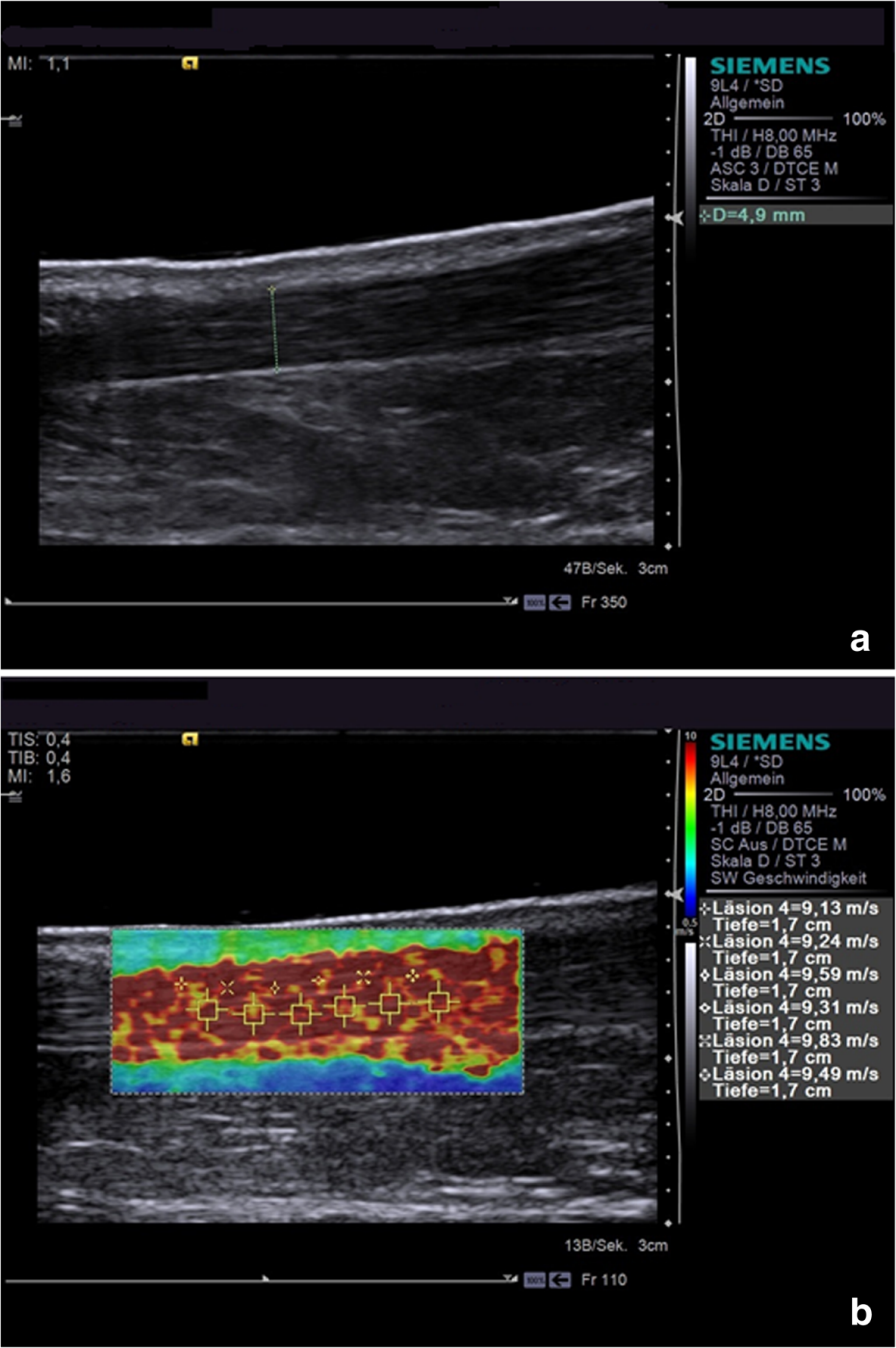
Depiction of the Achilles tendon using Acoustic Radiation Force Impulse. The figure shows an image obtained with the examination protocol. The transducer is held longitudinally and moved along the Achilles tendon in order to produce a sagittal view. The diameter of the tendon (**a**) and the elastographic measurement in VTIQ mode (**b**) were obtained in the same manner for each patient. We used a 9 L4 linear transducer with an Acuson S3000™ (Siemens Healthcare, Erlangen, Germany)

The study was carried out using the Siemens Acuson S3000 ™ (Siemens Medical Solutions USA, Inc.). We used a 9 L4 linear transducer for the B-scans and the elastographic measurements. To determine the location of the measurement, the examiner positioned the probe three fingerbreadths above an imaginary line through both malleoli. The probe was positioned longitudinally, corresponding to the direction of the tendon. In this way, the acoustic window provided a sagittal view of the organ. With positioning the probe longitudinally the examiner is able to cover more area of the Achilles tendon compared to the transversal position. The tendon was best visualized in the location described above, without interference of the gastrocnemius muscle or the calcaneus. Furthermore pathologies affecting the Achilles tendon seem to mostly manifest in this part, which is why we choosed to generate the normal values in this position. Measurements were only performed on the location described above. The shear wave velocity was determined in virtual touch image quantification (VTIQ) mode. The colour-coded elastogram, known as a box plot, is positioned in the organ under real-time B-mode guidance. The colour coding is explained by the legend on the right-hand side of the ultrasound scanner monitor. The colour spectrum ranges from blue areas corresponding to slow shear wave velocities, through greenish-yellow areas of intermediate velocity, to red areas indicating fast shear waves. Demonstration of the tissue hardness in VTIQ mode does not, however, follow qualitatively from the colour but through regions of interest (ROIs) that can be placed as desired within the box plot. The tissue stiffness is then quantitatively calculated in that ROI and depicted in m/s. In the present study, we used the following configuration for the B-mode image as well as the elastographic measurement:

The settings of the Acuson S3000 ™ were set as follows: The depth of the B-scan was 3.0 cm. The ARFI window had to be set to the smallest possible depth of 1.0 cm. The length of the window had to allow six ROIs to be placed adjacently in the middle of the tendon and was set accordingly. The ROIs were positioned along the longitudinal axis of the Achilles tendon (Fig. 1). The size of the ROI is predetermined by the Acuson S3000 and therefore not modifiable by the examiner. The positioning of the ROIs was guided by conventional B-Mode ultrasound (Fig. 1).

The team for each examination consisted of an examiner and a recorder, and the examination was completed in a single session. Nine postgraduates were involved in the measurements. They were trained specially for these ultrasonography measurements. The training involved over a month clinical practice performing ultrasound examinations on patients on a daily basis. After completing this month, every examiner was taught the ultrasound study protocol, as described above, by a DEGUM-certified medical doctor (German society for ultrasound in medicine). The protocol itself was developed by the examiners and the supervisors with the aim to generate reliable elastographic measurements while being practicable in the daily clinical routine. The supervisors were available to monitor the procedures and to provide further guidance in case there were any pathological findings.

We additionally tried to determine whether there was any relationship between the diameter of the tendon or the elastographic properties and various data extracted from the history of the subjects.

For this purpose, we collected anthropometric data such as height and weight, consumption of alcohol and tobacco, sporting activities and the period of fasting prior to the examination and looked whether there was a significant influence on our measurements.

### Phantom study

In order to determine the comparability of the data obtained by the nine postgraduates performing the examinations, we conceived the idea of additional measurements to test reliability. Three phantoms were fashioned: a piece of pig’s liver, a pig muscle, and two synthetic balls of different hardness. All examiners performed three repeated measurements in two separate series, and the data obtained were used to calculate the intra- and interclass correlation coefficient (ICC).

### Statistical analysis

Statistical analysis of the anonymised data was performed with SAS, Version 9.2 (SAS Institute Inc. Cary, NC, USA) statistics software. The data were first tested descriptively and the frequency, mean and standard deviation determined. The Shapiro-Wilk test was used to check for normal distribution. The *t*-test was used to determine differences in the means of normally distributed data, while the Wilcoxon rank sum test was used for data that did not show a normal distribution. The significance level was set at *p* < 0.05 (α = 0.05) for all tests.

The statistical analysis of the phantom study was performed with SPSS (IBM SPSS Statistics, Version 21). The data obtained were examined descriptively and the location parameters (mean, median) and dispersion parameters (standard deviation, minimum and maximum) presented.

The intra-rater (test-retest) and inter-rater reliability were validated with the two-factor intraclass correlation coefficient (ICC). Inter-rater reliability was determined from the means of the first measurement series in each case. The means of the three examination procedures were used to determine intra-rater reliability.

## Results

The study included 182 healthy volunteers, with data collected from both Achilles tendons in each volunteer, for a total of 364 tendonds examined. The study population consisted of 65 (35.7%) men and 117 (64.3%) women, with an overall mean age of 35.9 ± 15.2 years. The body mass index (BMI) was 23.2 ± 3 and the mean height was 171.1 ± 9.3 cm (Table 1). A mean ± standard deviation of 4.7 ± 0.9 mm was calculated for the diameter of the left Achilles tendon, compared to 4.8 ± 0.9 mm for the right.

**Table 1:** Demographic characterization of the study participants

Gender, n (%)	182 (100%)
Male	65 (35.7%)
Female	117 (64.3%)
Age, Mean ± STD	35.9 ± 15.2
17–24 years	56 (30.8%)
25–30 years	40 (22.0%)
31–40 years	25 (13.7%)
41–50 years	22 (12.1%)
51–60 years	22 (12.1%)
61–70 years	13 (07.1%)
71–85 years	4 (02.2%)
BMI, Mean ± STD	23.2 ± 3.0
BMI < 25	142 (78.0%)
BMI > 25	40 (22.0%)
BMI, subgroups	
BMI < 18.5	5 (2.8%)
BMI 18.5–24.9	137 (75.3%)
BMI 25.0–29.9	35 (19.2%)
BMI 30.0–34.9	4 (2.2%)
BMI 35.0–39.9	1 (0.6%)
Height, Mean ± STD	171.1 ± 9.3
150–160 cm	21 (11.5%)
161–170 cm	80 (44.0%)
171–180 cm	49 (26.9%)
181–190 cm	30 (16.5%)
191–200 cm	2 (01.1%)

*n* case number, *BMI* Body-Mass-Index, *STD* Standard deviation, *cm* centimeter

In the sagittal plane, the mean SWV was 9.09 ± 0.71 m/s on the left. The SWV was 9.17 ± 0.61 m/s on the right. There was a significant difference between the results for the left and right side (*p*-value < 0.05) (Table 2).

**Table 2:** Diameter and shear wave velocity of the Achilles tendon in sagittal view

Achilles tendon (*n* = 182)	Mean ± STD	95% CI	Min	Max	*p*-value
Diameter (mm)					
thickness on left	4.65 ± 0.91	4.62–4.88	2.50	7.40	< 0.05
thickness on right	4.75 ± 0.90	4.51–4.78	2.90	7.20	
Shear wave velocity (m/s)					
measurement on left	9.09 ± 0.71	8.98–9.19	5.72	9.99	< 0.05
measurement on right	9.17 ± 0.61	9.08–9.26	7.28	9.98	

*mean ± STD* mean ± standard deviation, *n* case number, *min* minimum value, *max* maximum value, *p*-value < 0.05 is significant, with a probability of error of 5%

We were unable to determine any significant difference between men and women for either the diameter or the shear wave velocity; nor did the subject’s age or BMI have any effect on either parameter (Tables 3, 4). Furthermore we found out, that neither the consumption of alcohol or tobacco nor the amount of sporting activities or fasting prior to examination had a significant influence on the diameter of the Tendon or the measured shear wave speed (Table 5).

**Table 3:** Influence of Age and BMI on the measurements performed on the Achilles tendon

Achilles tendon (*n* = 182)	Diameter (mm)		Shear wave velocity (m/s)	
	thickness on left	thickness on right	measurement on right	measurement on right
Age				
correlation	−0.11	−0.03	0.003	−0.08
coefficient				
*p*-value	0.13	0.66	0.97	0.26
BMI				
correlation	−0.03	0.0261	−0.08	0.03
coefficient				
*p*-value	0.66	0.73	0.26	0.67

*n* case number, *p*-value < 0.05 is significant, with a probability of error of 5%, *BMI* Body mass index

**Table 4:** Comparison of diameter and shear wave velocity measured in achilles tendon on women and men

Achilles tendon (*n* = 182)	Mean ± STD		*p*-value
	Men	Women	
Diameter (mm)			
thickness on left	4.60 ± 0.81	4.66 ± 0.96	0.7812
thickness on right	4.59 ± 0.81	4.83 ± 0.92	0.0925
Shear wave velocity (m/s)			
measurement on left	9.12 ± 0.69	9.06 ± 0.71	0.4985
measurement on right	9.19 ± 0.67	9.15 ± 0.56	0.2142

*n* case number, *p*-value < 0.05 is significant, with a probability of error of 5%

**Table 5:** Influence of Smoking, Alcohol, sport activity and Fasting prior to examination on the measurements performed on the Achilles tendon

Achilles tendon (*n* = 182)	Diameter (mm)		Shear wave velocity (m/s)	
	Left	Right	Left	Right
Consumption of tobacco				
correlation coefficient	−0.08	−0.09	0.04	0.06
*p*-value	0.27	0.22	0.58	0.41
Consumption of Alcohol				
correlation coefficient	−0.03	−0.08	−0.05	−0.03
*p*-value	0.67	0.31	0.52	0.69
Sport activity				
correlation coefficient	0.11	0.01	−0.09	−0.07
*p*-value	0.13	0.85	0.21	0.36
Fasting prior to examination				
correlation coefficient	−0.09	−0.30	−0.07	−0.12
*p*-value	0.64	0.10	0.71	0.52

*n* case number, *mm* millimeter, *m/s* meter per second, *p*-value < 0.05 is significant, with a probability of error of 5%, *Tobacco* Any given dose at least once per week, *Alcohol* Any given dose at least once per week, *Sport activity* More than one hour per week, *Fasting prior to examination* at least 6 h

The ICC determined for the inter-rater reliability was 0.931. The ICC for intra-rater reliability gave results between 0.857 and 0.979. Both results were calculated from measurements acquired in the phantom study.

## Discussion

In this prospective study of healthy volunteers we determined the normal values of the Achilles tendon assessed with ARFI-Elastography using the Acuson S3000. Our Results showed relatively fast shear waves with a mean of 9.09 ± 0.71 m/s on the left and 9.17 ± 0.61 m/s on the right Achilles tendon.

Looking more closely at elastographic studies on the Achilles tendon, we found that there is very little evidence for normal values. In our opinion, there are three factors that greatly limit the comparability of the available studies.

First, the parameters for characterising the elastography are usually given in either kPa or m/s. Aubry et al. (2015) showed that Young’s modulus – and therefore the results given in kPa – is not correctly calculated with respect to musculoskeletal tissues such as the Achilles tendon [8]. At the same time, they recommended that shear waves be presented in m/s and not in kPa. Accordingly normal values for the Achilles tendon that are given in kPa cannot be compared directly with results from studies working in m/s.

The second point concerns the comparability of the various ultrasonography device manufacturers, even if they all display the SWV in m/s. Trottmann et al. (2016) showed that the measurements obtained with the Aixplorer® were significantly higher than those with the Acuson S2000™ [5], when the same structures were examined. There are a few more studies comparing the Aixplorer® with the Acuson S2000™ [9, 10]. To the best of our knowledge, however, none of them has compared results from musculoskeletal structures or made a comparison with the S3000™.

The non-uniformity of the examination protocols for the Achilles tendon has to be taken into consideration as another factor, and the possible effects on the results discussed. In recent studies, the tendons have sometimes been examined in a relaxed state, maximally tensioned or in a neutral zero position. Comparing the SWV of the tendon under varying tensions, it was found that the lowest velocities were found with the Achilles tendon completely relaxed, i.e. measured with maximum plantar flexion. Dorsiflexion gave the highest values [8, 11].

In our protocol, the Achilles tendon was examined in one position only. The foot hung over the end of the examination table. The ankle joint was neither plantar flexed nor dorsiflexed and the foot was neither supinated nor pronated. We did not however use a device to maintain a certain position but relied on the patient and examiner, that the position was maintained during measurement. This might be a pitfall in our examination protocol, but we designed a study to produce standard values applicable in the daily clinical routine. A device to fixate the ankle is highly unlikely in such circumstances. The device would have to be standardized and available to all physicians performing ARFI-elastography.

The same goes for the applied pressure on the probe. As earlier described, no manual pressure was to be applied to the transducer during the examination, allowing only its own weight to be used. Again there was no device to quantify the applied pressure via the probe on to the tissue. Such device is currently not practicable in the daily routine when working with patients. It might be useful, that the manufacturer implements such a measurement directly in the probe. There yet has to be more evidence on how the manual applied pressure affects the elastographic measurement.

Positioning the transducer on the Achilles tendon also has effects on the measured SWV. As in the study by Fu et al., we characterised the Achilles tendon elastographically in the middle third of the tendon. The acoustic window, in the sagittal plane, lay about three fingerbreadths above the ankle. Compared with studies that examined multiple segments of the Achilles tendon, this may be a weakness in our protocol [3]. We choosed to examine the Achilles tendon at that location because it is easy sonographically depictable. There is no interference by muscle like in the proximal part of the tendon nor is it disturbed by the attachment to the calcaneus on its distal end. Furthermore, ruptures or tendinopathies seem to manifest mostly in the middle part of the tendon. According to a study by DeWall et al. (2014) the SWV values are highest just above the insertion on the calcaneus and decrease progressively as measurements are taken more proximally [12].

The choice of acoustic window can also have an effect on the shear waves. The shear waves appear to be much more rapid with a sagittal orientation of the transducer than in an axial elastogram of the tendon. Taking measurements on 326 healthy subjects, Fu et al. found the mean SWV measured axially to be 4.08 ± 0.54 m/s, while measurements with a sagittal orientation of the transducer gave a mean of 8.20 ± 1.07 m/s [7]. These findings were confirmed by another study, although the Acuson S3000 ™ was not used here [13]. The orientation of the fibres in the tendon is probably the determining factor for the different velocities. The Achilles tendon is an anisotropic organ. As such, the physical parameters change depending on the angle in which the ultrasound waves meet the tendon fibres [14–17]. Accordingly, shear waves spread faster along the fibres than transversely to them [8, 18]. In the sagittal plane, the mean SWV on the left was 9.09 ± 0.71 m/s; on the right, it was 9.17 ± 0.61 m/s.

In direct comparison with the similarly conceived study by Fu et al., the mean SWV in the sagittal plane in this case was found to be 8.20 ± 1.07 [7]. This study, as well as ours, found no significant difference between men and women. Furthermore, there were no significant differences between the age groups studied and the body mass index had no significant influence on the measured values. We consider these results very interesting. Although we examined subjects from 18 until the age of around 80 years, the shear wave speed did not show significant differences. Likewise Fu et al. described no significant age-related changes in the elastographic properties of the Achilles tendon. This might be because we only investigated healthy volunteers where the Tendon is intact. It could also mean that there are mechanisms which compensate for histologic age-related changes in the Achilles tendon to keep the elasticity on a similar level. Further investigations comparing histologic findings with elastographic measurements using the ARFI-technology could provide additional information.

The fact that we were not able to show a difference between men and women might suggest that any potential influence by hormones on the stiffness of tendonds is neglectable. We therefore have no reservations in including pregnant women in further studies.

Our examination protocol placed six ROIs for each measurement of the Achilles tendon in VTIQ mode. The mean for the corresponding side was calculated from the six values obtained. Fu et al. put three ROI’s in the sagittal plane and then three ROIs in the axial plane for further measurements. The SWVs determined in the two studies are very close. The difference might be explained by the different amount of ROIs used to acquire the mean value. Furthermore, ultrasound is an operator-dependent application and the results may vary. To determine whether the difference is because of regional differences, operator-dependency, the different amount of ROI’s or another reason, we suggest that further studies are conducted.

## Conclusion

In conclusion, with our study and that of Fu et al., there are two studies that have given similar values for the SWV and the diameter of the Achilles tendon. Taking the examination protocol into consideration, these values could serve as reference values for pathological changes in the tendons.

## Abbreviations

9 L4: Linear transducer (frequency 4,0–9,0 MHz)
ARFI: Acoustic Radiation Force Impulse
BMI: Body-Mass-Index
ICC: Intraclass correlation coefficient
kPa: Kilopascal
m/s: Meter per second
MHz: Megahertz
Mm: Millimeter
ROI: Region of Interest
SAS: Statistical Analysis System
SWE: Shear-wave-elastography
SWV: Shear-wave-velocity
VTIQ: Virtual Touch™ Imaging and Quantification
VTQ: Virtual Touch™ Tissue Quantification
